# Survival Analysis of Hospital Length of Stay of COVID-19 Patients in Ilam Province, Iran: A Retrospective Cross-Sectional Study

**DOI:** 10.3390/jcm12206678

**Published:** 2023-10-23

**Authors:** Khalil Momeni, Mehdi Raadabadi, Jamil Sadeghifar, Ayoub Rashidi, Zahra Toulideh, Zahra Shoara, Morteza Arab-Zozani

**Affiliations:** 1 Department of Health Economics and Management, School of Health, Ilam University of Medical Sciences, Ilam 6931851147, Iran; 2 Health Policy and Management Research Center, School of Public Health, Shahid Sadoughi University of Medical Sciences, Yazd 8916978477, Iran; 3 Health and Environment Research Center, Ilam University of Medical Sciences, Ilam 6931851147, Iran; 4 Department of Health Economics and Management, Ilam University of Medical Sciences, Ilam 6931851147, Iran; 5 Social Determinants of Health Research Center, Birjand University of Medical Sciences, Birjand 9717853577, Iran

**Keywords:** survival analysis, Cox regression, hospital length of stay, COVID-19, Ilam, Iran

## Abstract

Knowledge of the length of hospitalization of patients infected with coronavirus disease 2019 (COVID-19), its characteristics, and its related factors creates a better understanding of the impact of medical interventions and hospital capacities. Iran is one of the countries with the most deaths in the world (146,321 total deaths; 5 September 2023) and ranks first among the countries of the Middle East and the EMRO. Analysis of confirmed COVID-19 patients’ hospital length of stay in Ilam Province can be informative for decision making in other provinces of Iran. This study was conducted to analyze the survival of COVID-19 patients and the factors associated with COVID-19 deaths in the hospitals of Ilam Province. This is a retrospective cross-sectional study. Data from confirmed COVID-19 cases in Ilam Province were obtained from the SIB system in 2019. The sample size was 774 COVID-19-positive patients from Ilam Province. Measuring survival and risk probabilities in one-week intervals was performed using Cox regression. Most patients were male (55.4%) and 55.3% were over 45 years old. Of the 774 patients, 87 (11.2%) died during the study period. The mean hospital length of stay was 5.14 days. The median survival time with a 95% confidence interval was four days. The probability of survival of patients was 80%, 70%, and 38% for 10, 20, and 30 days of hospital stay, respectively. There was a significant relationship between the survival time of patients with age, history of chronic lung diseases, history of diabetes, history of heart diseases, and hospitalization in ICU (*p* < 0.05). The risk of dying due to COVID-19 disease was higher among men, older age groups, and patients with a history of chronic lung diseases, diabetes, and heart disease. According to the results, taking preventive measures for elderly patients and those with underlying conditions to prevent the infection of COVID-19 patients is of potential interest. Efficiency in the management of hospital beds should also be considered.

## 1. Introduction

Coronavirus disease 2019 (COVID-19) is an infectious disease caused by severe acute respiratory syndrome coronavirus 2 (SARS-CoV-2) [1]. Coronaviruses were first identified in the 1930s, infecting only animals. The human type of these viruses was determined in 1960 [2]. These viruses have mutated from simple cold symptoms to acute respiratory problems. The first case of a COVID-19 patient was reported in Wuhan, China, at the end of 2019, and then it spread in China and around the world until the World Health Organization (WHO) declared it a pandemic [3,4,5].

COVID-19 is commonly asymptomatic [6]. Many of these asymptomatic patients recover over time without the need for medical care and hospitalization [7]. However, a significant number of these patients show multiple symptoms. Patients with COVID-19 present several manifestation including pneumonia, acute respiratory failure [8], cardiovascular symptoms [9,10], renal failure [11], hematological symptoms [12], and cutaneous disorders [8,13]. These patients usually need to be hospitalized and receive specialized care [14]. Having old age along with other diseases such as a history of hypertension, diabetes, obesity, and smoking significantly increase the risk of contracting this disease and the need for hospitalization [15,16,17]. Also, the characteristics of COVID-19 can be vary depending on the demographic and epidemiological characteristics of that country [15]. Iran is one of the countries with the most deaths in the world (146,321 total deaths; 5 September 2023) and ranks first among the countries of the Middle East and the EMRO region of the World Health Organization (WHO).

Knowing the duration of hospitalization of patients, characteristics of patients, and factors related to duration of hospitalization creates a better understanding of the impact of medical interventions and hospital capacities on patients. Hospital length of stay (LOS) is a clinical metric that measures the length of time elapsed between a patient’s hospital admittance and discharge. The LOS of these patients depends on several factors, including the duration from initial exposure until the appearance of the first symptoms of the disease, the duration from appearance of initial symptoms until the time of admission to the hospital, and specific background factors of each context [18,19,20]. The COVID-19 pandemic has led to a surge in hospital admissions worldwide. The LOS of COVID-19 patients in hospitals has become a significant concern for healthcare providers, policymakers, and patients themselves [21].

Various studies have been conducted and published in this area. A study conducted by Guan et al. (2020) analyzed the LOS of 1590 COVID-19 patients in Wuhan, China. They found that the median LOS was 12 days, with a range of 4 to 41 days. The study also reported that LOS was longer for patients who required mechanical ventilation or had comorbidities such as hypertension or diabetes [22]. Another study by Grasselli et al. (2020) analyzed data from 1591 COVID-19 patients admitted to 72 Italian intensive care units (ICUs). The study reported that the median LOS for ICU patients was 15 days, with a range of 1 to 60 days. The study also found that older age, higher Sequential Organ Failure Assessment (SOFA) score, and the need for mechanical ventilation were associated with longer ICU stays. Also, based on results of this study, the 28-day mortality rate was 26.9% [23]. Also, Wang et al. (2020) analyzed the LOS of 138 COVID-19 patients in Wuhan, China. The study reported that the median LOS was 14 days, with a range of 7 to 41 days. The study also found that older age, higher SOFA score, and the presence of comorbidities were associated with longer LOS. Based on results of this study, the median time from symptom onset to discharge was 25 days, with a range of 10 to 51 days [24]. Salinas-Escudero et al. conducted a survival analysis of COVID-19 patients in the Mexican population. Their analysis was based on the 16,752 confirmed cases of COVID-19. Based on results of this study, factors including male sex, older age, chronic kidney disease, pneumonia, hospitalization, intensive care unit admission, intubation, and health care in public health services were independent factors increasing the risk of death due to COVID-19. Also, the general mortality rate was 4.94 (95% CI: 4.70–5.19) per 1000 person-year [25]. Kaso et al. conducted a survival analysis of COVID-19 patients in a hospital based study in Ethiopia. The overall mortality rate for this study was 6.35 cases per 1000 person-days. Older age (>46), chronic obstructive pulmonary disease, chronic kidney disease, admission to an intensive care unit, and being on intranasal oxygen care were independent risk factors of death from COVID-19 disease and increased this risk more than their counterparts [26]. Muyinda et al. reported the results of a cohort study in Uganda in 2021. Based on their results from six hospitals, the mortality rate for this study was 30.7 deaths per 1000 person days. Also, the medical survival time was 33 days. Several factors associated with time to COVID-19 death included age over 60 years, malaria test at admission, and admission to public hospitals [27]. Another study by Bobdey et al. investigated the mortality and survival of COVID-19 patients in a tertiary-care hospital in Maharashtra, India. Based on results of this study, a significantly higher risk of mortality was observed in elderly patients (age > 60 years), patients with multiple comorbidities, and patients requiring oxygen therapy [28]. A systematic review by Wynants et al. (2020) analyzed data from 24 studies that reported on the LOS of COVID-19 patients. The review found that the median LOS ranged from 7 to 26 days across studies. The review also reported that patients who required ICU admission had longer LOS than those who did not require ICU admission [29].

Due to the significant increase in mortality and the speed of spread of this disease, various methods have been used to check and predict the survival of patients infected with this virus. Usually, these methods are developed and applied based on the symptoms and clinical parameters of the patients. Survival analysis is a model for determining when a given event will occur. It is possible to calculate the probability of survival and the effect of each of the characteristics of the patients such as the symptoms of the patients on these probabilities using survival analysis [30,31]. There are various models for survival analysis, the most common of which is the Cox model, which uses regression models to analyze survival [32,33].

To the best of our knowledge, no analysis has been yet documented from Ilam Province examining the survival of COVID-19 and associated risk factors. Therefore, this study aimed to analyze the survival of COVID-19 patients based on their length of hospital stay. Also, we identified the factors associated with the survival time of COVID-19 patients in Ilam Province.

## 2. Materials and Methods

This retrospective cross-sectional study was conducted using the medical records of 774 laboratory-confirmed cases of COVID-19 admitted to intensive care units (ICUs) and infectious disease departments of hospitals affiliated with the Ilam University of Medical Sciences. Data from confirmed COVID-19 cases in Ilam Province were obtained from the SIB system. The SIB system is an integrated health system that includes all data related to all health outcomes including COVID-19 data (https://sib1.medilam.ac.ir/, Accesses date: 20 February 2021). The database included all positive, negative, and suspected cases of COVID-19.

Ilam Province is one of the 31 provinces of Iran and the 22nd largest province of Iran. This province is located in the west of Iran with an area of about 20,164.11 square kilometers. This province shares a 425 km border with Iraq. Also, this province neighbors Kermanshah, Lorestan, and Khuzestan provinces. Ilam is the largest city and also the capital of Ilam Province. Based on a 2016 census, the population of this province was about 580,158 people in 159,310 households, the least populated province in Iran. The predominant language in Ilam Province is Kurdish but the province has a diverse linguistic presence because of its position in the transitional zone between Kurdish and the Southwestern Iranian language blocs. At the time of data collection, Ilam Province ranked 26th in the country in terms of number of deaths.

Data were collected from 20 February 2020 to 20 February 2021. All patients tested positive using a polymerase chain reaction (PCR) SARS-CoV-2 assay of nasopharyngeal swab specimens. Sampling in this study was conducted by a census method and the information of all COVID-19 patients in this period was examined.

The inclusion criteria were the positivity of the samples through PCR testing in patients hospitalized in the ICU and infectious wards of Ilam City and the completeness of the information recorded in the medical records system. PCR is a test to detect genetic material from a specific organism, such as a virus. The PCR test used for detecting COVID-19 patients is a molecular test that analyzes upper respiratory specimens for genetic material (ribonucleic acid or RNA) of SARS-CoV-2. The cases that had incomplete data or no suitable data at all were excluded from the study. Twenty-three records were eliminated as the data regarding death were missing.

The data collection tool was a researcher-made checklist based on the available information in the dashboard for COVID-19 patients. This checklist included demographic (age, gender), diagnostic (result of PCR test), and clinical information of the patients, including history of underlying diseases (chronic lung disease, chronic neurological disease, kidney disease, liver disease, diabetes, and cardiovascular disease), history of using mechanical ventilation (yes or no), and length of stay (mean hospital length of stay). Having or not having an underlying disease was based on the doctor’s diagnosis and based on international guidelines and was extracted from the patient’s record.

The outcome variable of interest for our study was time to death since COVID-19 symptom onset. The socio-demographic factors, health-related factors, and comorbidity condition factors were treated as independent variables. The survival analysis and the Cox regression model included dichotomic values for sex, chronic lung disease, chronic neurological disease, kidney disease, liver disease, diabetes, cardiovascular disease, and mechanical ventilation. Overall, our analysis considers the following variables: age, sex, and comorbidities such chronic lung disease, chronic neurological disease, kidney disease, liver disease, diabetes, cardiovascular disease, and mechanical ventilation during the onset of symptoms, hospitalization, and death.

### Statistical Analysis

Mean and median survival times were used to describe the data. The study outcome was survival rate. Cox regression analysis was used to determine factors associated with death among hospitalized COVID-19 patients. To perform the Cox regression, first, the necessary preconditions for the model, i.e., the randomness of the censoring of the observations and the constancy of the relative risk over time in the study groups, were checked. The randomness of the censoring of observations was achieved by plotting the censored data against time and constancy of the relative risk by drawing a log-minus-log graph and checking for absences of intersections in the graph. The Kaplan–Meier method was used to plot survival curves. Also, survival functions were obtained by the Kaplan–Meier method. All collected data were entered, coded, and analyzed using Statistical Package for Social Science (SPSS) software Version 21, considering a significance level of 5%.

The Iran National Committee for Ethics in Biomedical Research at the Ilam University of Medical Sciences approved ethical clearance, and the Institutional Review Board (IRB) waived the informed consent procedure for this study. Since the study was conducted through the SIB system of medical records, consent to participate was waived. Individual patients were not harmed, and the data analyzed were used for this study only.

## 3. Results

Most patients were male (55.4%), and 55.3% were over 45 years old. Of 774 patients, 87 (11.2%) died during the study period. The minimum and maximum LOS values were 1 day and 32 days, respectively. The mean hospital length of stay of patients was 5.14 days and the median length of stay was 4 days. A total of 16.4% of patients were hospitalized in intensive care units. Regarding underlying diseases, 17.7% had cardiovascular diseases, 13.8% had diabetes, 9.6% had kidney diseases, 8.8% had chronic neurological diseases, and 9.3% had chronic lung diseases.

**Figure 1:**
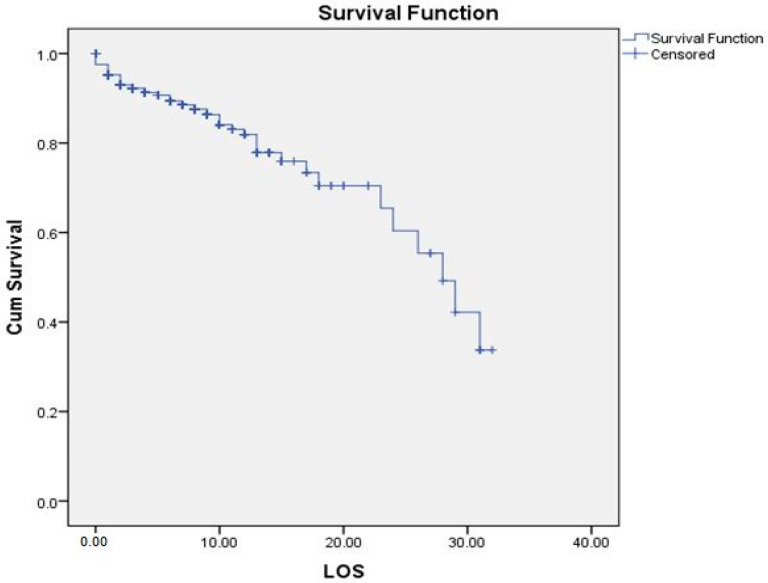
Probability of survival of COVID-19 patients according to time. LOS: length of stay; Cum Survival: Cumulative survival.

According to Figure 1, the probability of survival of patients was 80%, 70%, and 38% for 10, 20, and 30 days of hospital stay, respectively.

### Factors Associated with the Rate of Death among COVID-19 Patients

Multiple Cox regression results showed that patients younger than 45 years had a 63% lower risk of death compared to patients older than 45 years (hazard ratio: HR = 0.37, *p* < 0.001). Also, women had a 32% lower risk of death than men. Patients with a history of chronic lung diseases, diabetes, and heart disease had a higher risk of death compared to patients without a history. Patients with a history of chronic lung diseases, diabetes, and heart diseases compared to patients without a history had 154% (HR = 2.54, *p* = 0.001), 36% (HR = 1.36, *p* = 0.021), and 98% (HR = 1.49, *p* < 0.03) higher risks of death, respectively. Patients hospitalized in intensive care units had a 275% higher risk of death than others (HR = 3.75, *p* = 0.001) (Table 1).

The results of regression analysis showed that increasing age by one year increases the chance of death by 3% (*p* = 0.001). Also, an increase of one day in hospital length of stay increases the chance of death by 5% (*p* = 0.001). Having underlying diseases increases the mortality rate as follows: cardiovascular disease, 2.2 times; chronic neurological disease, 3.3 times; chronic lung disease, 5.4 times; kidney diseases, 2.7 times; liver diseases, 2.5 times; and diabetes, 2.6 times. In addition, patients who were hospitalized in intensive care units had a lower chance of survival (Table 2).

## 4. Discussion

This study aimed to analyze the survival of COVID-19 patients based on their length of hospital stay. Also, we identified the factors associated with the survival time of COVID-19 patients in Ilam Province. Our study demonstrated that 11.2% of the study participants died during the study period. The mean survival time was 5.14 days. The median survival time with a 95% confidence interval was four days. The probability of survival of patients was 80%, 70%, and 38% for 10, 20, and 30 days of hospital stay, respectively. There was a significant relationship between the survival time of patients and age, history of chronic lung diseases, history of diabetes, history of heart diseases, and hospitalization in ICUs (*p* < 0.05). The risk of dying due to COVID-19 disease was higher among men, older age groups (>45 years), and patients with a history of chronic lung diseases, diabetes, and cardiovascular disease. Also, patients hospitalized in intensive care units had a 275% higher risk of death than others (HR = 3.75, *p* = 0.001)

Based on the results of the present study, the mean length of stay of hospitalized patients with COVID-19 was 5.14 days, and the median was 4 days. Lapidus et al. (2020) reported a length of stay of 21 days for COVID-19 patients in ICUs, which was much higher than the estimates reported in the present study [34]. The conservative and unbiased estimates of the average length of stay (ALOS) in ICUs show that a higher burden of ICU bed occupancy is assumed than in COVID-19 prediction models [34]. Meanwhile, in the study by Vekaria et al. (2021), the average length of stay of patients hospitalized in ICUs was calculated as 8 days. The results of their study were higher than the results of our study. The reason may be due to the larger number of elderly people in the study [35]. Boëlle et al. (2020) also analyzed 1321 patients admitted to hospitals in the north and east of France. They estimated that the length of stay of COVID-19 patients in the hospitals was 15.9 days and the mortality rate was 20% [36]. Also, Guan et al. (2020) reported a median LOS of 12 days, with a range of 4 to 41 days [22]. Grasselli et al. (2020) analyzed data from 1591 COVID-19 patients admitted to 72 Italian intensive care units (ICUs). The study reported that the median LOS for ICU patients was 15 days, with a range of 1 to 60 days [23]. Most studies have reported these values as higher than in our study, which could be due to differences in demographic characteristics, comorbidities, and differences in clinical manifestations of patients.

The majority of patients were male (55.4%) and 55.3% were over 45 years old. Fried et al. (2021) examined a total of 11,721 patients hospitalized in 245 hospitals in the United States of America. Among them, the majority were over 60 years old and most of them were men [37]. Kaso et al. reported the results of 422 COVID-19 patients’ records. In their study, the mean age of the participants was 41.06 ± 20.61 years. The majority of the participants were males (61.4%), and around three-fifths (60.4%) of them were from urban areas [26]. In the study by Muyinda et al., the median age of participants was 52 years, with an interquartile range (IQR) of 34 to 67 years. Slightly more than half of the participants were female, 555/1038 (53.5%) [27]. Also, in the systematic review study by Romero-Sánchez et al. (2020), 841 hospitalized patients with COVID-19 were investigated, among which, the median age of the majority of hospitalized patients was 66.4 years and 56.2% of the patients were men [38]. In a retrospective cohort study by Casas-Rojo et al. (2020), it was also shown that among 15,111 patients hospitalized in 150 Spanish hospitals, 57.2% were men and the average age of hospitalized patients was 69.4 years [39]. Therefore, it can be argued that the elderly and men are at a higher risk of contracting this disease.

Based on the Cox regression results in the present study, some demographic and epidemiological factors affected the hospital length of stay of patients. Patients younger than 45 years had a lower risk of death compared to patients older than 45. In line with the results of the present study, the hospital length of stay for people over 46 years of age was longer in the studies of Thai et al. (2020) [18]. Also, Kaso et al. conducted a survival analysis of COVID-19 patients in a hospital-based study in Ethiopia. Based on results of their study, which are also consistent with our results, older age (>46) was an independent risk factor that increased the risk of death from COVID-19 disease more than its counterparts [26]. The results of studies conducted in other countries also showed that hospital length of stay increased with the increasing age of patients [8,40,41]. Usually, if the severity of disease and underlying diseases is higher in the elderly, then these people need more care. In some studies, this age is reported to be 60 years and older, which is not consistent with our results. For example, studies by Muyinda et al. and Bobdey et al. reported that a significantly higher risk of mortality was observed in elderly patients (age > 60 years) [27,28]. In our study, patients with a history of chronic lung diseases, diabetes, and cardiovascular diseases had a higher risk of death compared to patients without a history. According to the results of other studies, the probability of becoming sick and dying is higher in COVID-19 patients who have underlying diseases such as cardiovascular diseases, diabetes, chronic respiratory diseases, high blood pressure, and cancer [8,42,43]. Also, Bobdey et al. investigated the mortality and survival of COVID-19 patients in a tertiary-care hospital in Maharashtra, India, and showed that a significantly higher risk of mortality was observed in patients with multiple comorbidities and in patients requiring oxygen therapy [28]. Kaso et al. also confirmed our results. Based on a survival analysis of COVID-19 patients in a hospital-based study in Ethiopia, chronic obstructive pulmonary disease and chronic kidney disease were independent risk factors increasing risk of death from COVID-19 disease more than their counterparts [26].

Regarding gender, the risk of death was higher in men than in women. The results of a study showed that men die more than women [8], although most of the studies conducted among COVID-19 patients did not show a significant difference between mortality and disease outcomes according to gender [44,45,46]. In line with our results, Salinas-Escudero et al. reported that male sex was an independent factor increasing the risk of death due to COVID-19 [25]. The higher hospital length of stay of men compared to women can be related to the higher number of men infected with diseases.

This study has some limitations. This study is based on data from Ilam Province and may not represent national pictures. In addition, as this study is based on a retrospective review of medical records, it may not display all factors that may predict variation in survival probabilities. The hospital staff also have important duties. Also, as our data collection was limited, sub-analyses stratified by clinical and laboratory baseline conditions, or among those admitted to ICUs, were not performed. There is a large difference in the length of hospitalization between individual patients with a range of variation of 1–32 days. Unfortunately, due to the lack of access to relevant data and laboratory parameters, the effect of applied therapy on outcome was not investigated.

We will try to include these items in future articles resulting from the plan.

## 5. Conclusions

Based on our results, the mean survival time was 5.14 days. Also, the risk of dying due to COVID-19 disease was higher among men, older age groups, and patients with a history of chronic lung diseases, diabetes, and heart disease. Reducing the length of stay of COVID-19-hospitalized patients and management of hospital beds are important tasks of hospital managers and policymakers and require careful planning. A detailed medical history from COVID-19 patients can be effective in providing appropriate, adequate, and timely medical services, reducing mortality and reducing LOS. Considering the fact that underlying diseases and comorbidities can increase the risk of mortality and prolong LOS, the knowledge of hospital staff about these cases can help the treatment process and further care for this category of patients. By learning from the experience of the COVID-19 pandemic, it seems that focus on mitigation campaigns for at-risk populations with more fatal consequences can be useful. Also, establishment of public health campaigns aimed at reducing the prevalence of obesity, diabetes, hypertension, and their comorbidities is essential during such pandemics and after them.

## Figures and Tables

**Table 1:** Survival of COVID-19 patients based on multiple Cox regression.

Variable		HR	95% CI	*p*-Value
Gender	Female	0.68	0.44, 1.06	0.04
	Male	1	-	
Age	<45 years	0.37	0.22, 0.64	0.001
	>45 years	1	-	
Chronic lung disease	Yes	2.54	1.56, 4.13	0.001
	No	1	-	
Chronic neurological disease	Yes	1.54	0.90, 2.61	0.111
	No	1	-	
Kidney disease	Yes	1.53	0.90, 2.60	0.109
	No	1	-	
Liver disease	Yes	1.24	0.46, 1.24	0.462
	No	1	-	
Diabetes	Yes	1.36	0.83, 2.24	0.021
	No	1	-	
Cardiovascular disease	Yes	1.49	0.93, 2.39	0.03
	No	1	-	
Mechanical ventilation	Yes	3.75	2.07, 6.77	0.001
	No	1	-	

**Table 2:** The effective epidemiological factors on mortality of COVID-19 patients based on regression analysis.

	B	S.E.	Wald	df	Sig.	Exp(B)	95% C.I. for EXP(B)	
							Lower	Upper
Age	0.030	0.006	25.524	1	0.001	1.030	1.018	1.042
LOS	0.005	0.021	0.056	1	0.040	1.055	0.964	1.048
Sex	0.419	0.237	3.139	1	0.076	1.521	0.956	2.418
Chronic lung disease	1.859	0.278	44.622	1	0.000	6.420	3.720	11.078
Chronic neurological disease	1.466	0.293	25.111	1	0.000	4.333	2.442	7.688
Kidney disease	1.613	0.288	20.910	1	0.000	3.730	2.122	6.557
Liver disease	1.267	0.312	16.474	1	0.000	3.549	1.925	6.543
Diabetes	1.214	0.260	21.767	1	0.000	3.677	2.022	5.606
Cardiovascular disease	1.171	0.246	22.597	1	0.000	3.225	1.990	5.226
Inpatient in ICU	1.673	0.340	24.198	1	0.00	5.330	2.736	10.381

## Data Availability

Due to privacy and ethical concerns, the data that support the findings of this study are available on request from the first author (U.K.).
